# Evaluating the Role of PhasixST™ Mesh in Laparoscopic Repair of Large Hiatal Hernias: Surgical Technique and Comprehensive Review of the Literature

**DOI:** 10.3390/jcm14238316

**Published:** 2025-11-23

**Authors:** Lazaros Kourtidis, Katerina Neokleous, Konstantina Spyridaki, Dimitra Ntrikou, Michail Lazaris, Theodora Choratta, Melina Papalexandraki, Eleni Markaki, Marilena Tsivgouli, Athanasios Kalligas, Ioannis Papazacharias, Charalampos Theodoropoulos, Dimitrios Margaritis, Panagiotis Dikaiakos, Efstratios Kouroumpas, Christos Iordanou, Georgios Ayiomamitis

**Affiliations:** 1st Surgical Department, Laparoscopic Unit, General Hospital of Piraeus “Tzaneio”, 18536 Piraeus, Greece; katerneo1995@gmail.com (K.N.); konstantinasp.gr@gmail.com (K.S.); dimitra.ntrikou@gmail.com (D.N.); mixlazar@gmail.com (M.L.); theodora.choratta@yahoo.com (T.C.); melinapapalex@gmail.com (M.P.); elenimarkaki97@gmail.com (E.M.); mtsivgouli@gmail.com (M.T.); thkalligas@me.com (A.K.); giannis92pap@gmail.com (I.P.); chtheodoropoulos91@gmail.com (C.T.); d.mrg@hotmail.com (D.M.); dikeakosp@gmail.com (P.D.); ekouroumpas@gmail.com (E.K.); chriordanou@yahoo.gr (C.I.); agiogeo@gmail.com (G.A.)

**Keywords:** hiatal hernia, laparoscopic repair, mesh, PhasixST™

## Abstract

The application of bioabsorbable PhasixST™ mesh in the laparoscopic repair of large hiatal hernias has emerged as a promising strategy to address the limitations associated with permanent synthetic meshes, particularly the risk of mesh-related complications and long-term morbidity. Recent studies have demonstrated that PhasixST™ mesh, composed of poly-4-hydroxybutyrate (P4HB), is slowly absorbed over 12 to 18 months, providing a scaffold that supports native tissue integration and healing during the critical postoperative period. This gradual absorption profile may confer an advantage over more rapidly degrading bioabsorbable meshes, as it allows for more robust tissue ingrowth and potentially enhances the durability of hiatal reinforcement. The association between P4HB mesh use and low recurrence rates has been highlighted, with an average of 2.82 hernia recurrences per 100 patients within one year, and no mesh-related complications in the current literature. The surgical technique for PhasixST™ mesh placement involves meticulous crural reinforcement, with careful attention to mesh orientation and fixation to minimize the risk of migration or erosion. The primary objective is to restore the anatomical integrity of the hiatus, reduce the size of the defect, and prevent recurrence, while minimizing perioperative morbidity.

## 1. Introduction

Hiatal hernia is a pathological condition characterized by the abnormal protrusion of abdominal contents, most commonly the stomach, through the esophageal hiatus of the diaphragm into the thoracic cavity. The anatomical disruption at the esophageal hiatus can lead to a wide spectrum of clinical manifestations, ranging from asymptomatic cases to severe gastroesophageal reflux disease (GERD), dysphagia and acute mechanical complications. Large hiatal hernias represent a significant clinical burden due to their higher recurrence rates, greater anatomical distortion, and increased risk of life-threatening complications such as volvulus, obstruction, and ischemia. These factors make their management more challenging compared to smaller defects [1,2].

The classification of hiatal hernias is traditionally based on the anatomical relationship between the gastroesophageal junction (GEJ), the stomach, and the diaphragmatic hiatus. The most widely accepted system divides hiatal hernias into four main types. Type I, or sliding hiatal hernia, is the most prevalent form and involves the upward displacement of the GEJ and a portion of the stomach into the thoracic cavity. In this type, the GEJ migrates above the diaphragm, while the gastric fundus remains below it. Type II, or paraesophageal hernia, is characterized by herniation of the gastric fundus alongside the esophagus through the hiatus, with the GEJ remaining in its normal intra-abdominal position. Type III represents a combination of types I and II, where both the GEJ and a significant portion of the stomach herniate into the thorax. Type IV is defined by the herniation of other abdominal organs, such as the colon or small intestine, in addition to the stomach, through a markedly enlarged hiatus [3,4,5]. Among these, large type III and IV hernias carry the highest morbidity, often presenting with obstructive symptoms or respiratory compromise, particularly in elderly and comorbid populations. The size and location of the herniated contents, as well as the degree of disruption of the diaphragmatic crura, are critical factors influencing the risk of recurrence and the choice of surgical technique [3,5,6].

The clinical presentation of paraesophageal hernias varies, with many patients remaining asymptomatic, while others may experience symptoms ranging from reflux and dysphagia to acute complications such as gastric volvulus or ischemia. The risk of severe complications, including strangulation and perforation, is relatively low in asymptomatic patients, but if an acute event occurs, it is associated with increased morbidity and mortality, particularly if emergent surgical intervention is required. The decision to proceed with surgical repair is determined by the presence and severity of symptoms, patient comorbidities, and the risk for perioperative complications. Elective repair is generally favored over emergent intervention due to improved postoperative outcomes, a fact that has been further supported by the adoption of minimally invasive surgical techniques [7].

The pathophysiology underlying hiatal hernia formation is multifactorial, involving congenital or acquired weakness of the phrenoesophageal membrane, increased intra-abdominal pressure, and age-related changes in connective tissue integrity. The surgical management of large hiatal hernias is a complex and evolving field and the surgical approach aims to restore the normal anatomy by reducing the herniated stomach and other organs into the abdominal cavity, decreasing the size of the hiatal defect, and reinforcing the crural closure to prevent recurrence [3,4]. The choice of reinforcement material, such as bioabsorbable mesh, is determined by the hernia type, defect size, and patient-specific risk factors. Recent literature emphasizes the importance of standardized definitions and classifications to ensure consistency in reporting outcomes and to facilitate the comparison of different surgical techniques and materials.

Despite improving techniques, recurrence remains a notable challenge, with reports indicating recurrence rates as high as 42% to 66% following large hiatal hernia repair using primary suture cruroplasty alone [8]. Recurrence rates are influenced by the selection of repair techniques and the type of mesh utilized for crural reinforcement. Permanent synthetic meshes have demonstrated efficacy in reducing recurrence, but their use is tempered by the risk of mesh-related complications, including erosion, infection, and chronic pain. Studies have reported recurrence rates of 30.6% after long-term follow-up with permanent synthetic meshes in complex hernia cases, while other investigations have observed lower rates, such as 19% after 20 months, highlighting variability based on patient selection and surgical context [9].

These limitations have driven interest in mesh reinforcement. Traditional suture-based cruroplasty, while effective in many cases, is associated with a substantial risk of recurrence, especially when the hiatal defect exceeds a certain size threshold, due to the inherent weakness of the diaphragmatic crura and the constant dynamic forces exerted by the esophagus and stomach [3,7]. Several studies have indicated that the size of the esophageal hiatus is a critical determinant of recurrence risk, with defects greater than 5 cm^2^ being particularly prone to failure when repaired with sutures alone [10]. This observation has led to the consideration of mesh reinforcement as a means to provide additional structural support to the crural closure, thereby reducing the likelihood of early and late recurrences. Mesh reinforcement aims to distribute tension more evenly and strengthen the repair against repetitive biomechanical stresses. The use of mesh is especially advocated in large hiatal hernias, where the tissue quality may be compromised and the risk of suture pull-through is heightened [3,11]. Permanent synthetic meshes, while effective in reducing recurrence rates, have been associated with significant complications, including mesh erosion, chronic pain, and esophageal stricture formation, prompting the search for alternatives [12].

In this context, bioabsorbable meshes such as PhasixST™ have emerged as promising solutions. Designed to provide temporary reinforcement during the critical healing period while gradually resorbing, PhasixST™ aims to preserve the benefits of mesh support while reducing long-term foreign body complications. Early evidence suggests favorable recurrence profiles and low complication rates, attributed to the mechanical properties and biocompatibility of poly-4-hydroxybutyrate (P4HB) [1]. The association between P4HB mesh use and low recurrence rates has been highlighted, with an average of 2.82 hernia recurrences per 100 patients within one year, and no mesh-related complications in the current literature [1,2]. Nevertheless, long-term data remain limited, and the optimal integration of bioabsorbable materials into hiatal hernia repair continues to evolve [3,6].

Despite advances in minimally invasive surgery and the use of mesh reinforcement, a significant knowledge gap remains regarding the optimal method for achieving a durable hiatal repair while minimizing long-term complications. Primary suture cruroplasty alone is associated with substantial recurrence, particularly in large defects, while permanent synthetic meshes—although effective in reducing recurrence—carry well-documented risks of erosion, stricture, chronic pain, and long-term foreign body reactions. These limitations underscore the need for alternative strategies that provide adequate short-term mechanical support without the long-term risks inherent to permanent implants. Bioabsorbable meshes, such as PhasixST™, have emerged as promising candidates to fill this gap, yet long-term comparative data remain limited. Their potential to balance reinforcement durability with improved safety highlights an important area of ongoing investigation and justifies further exploration in the present review.

## 2. Biochemical Characteristics and Advantages of PhasixST™ Mesh

Biosynthetic and bioabsorbable meshes have emerged as important alternatives to traditional permanent synthetic materials in the context of hiatal hernia repair. These meshes are designed to provide temporary mechanical support to the crural closure, allowing for tissue integration and subsequent gradual resorption, thereby potentially reducing the long-term complications associated with nonabsorbable meshes such as fibrosis, chronic pain, and erosion. The rationale for their use is grounded in the hy-pothesis that a scaffold which is eventually absorbed by the body may minimize the risk of excessive scarring and mesh-related morbidity, while still reinforcing the repair during the critical period of tissue healing [13,14,15].

PhasixST™ mesh is a bioabsorbable material specifically engineered for soft tissue reinforcement. Its primary constituent is poly-4-hydroxybutyrate (P4HB), a naturally derived polymer synthesized via fermentation by Escherichia coli bacteria. The polymerization process yields a high-molecular-weight, linear polyester that is both biocompatible and bioresorbable, distinguishing it from traditional permanent synthetic meshes. The mesh is designed to remain structurally intact for up to two years in vivo, providing a prolonged period of mechanical support during the critical phases of tissue healing and remodeling [1]. P4HB exhibits a semi-crystalline structure, which imparts a balance between flexibility and tensile strength. This characteristic is essential for withstanding the dynamic mechanical forces at the esophageal hiatus while minimizing the risk of mesh-related complications such as erosion or chronic inflammation.

Bioabsorbable meshes such as PhasixST™ are engineered to provide temporary mechanical support to the hiatal closure while facilitating gradual integration with host tissue. Biomechanical studies have demonstrated that bioabsorbable meshes can offer sufficient suture retention strength and durability during the initial postoperative period, which is crucial for the success of the repair [16]. The process of bioabsorption is characterized by a controlled degradation of the polymeric scaffold, which is designed to coincide with the progressive ingrowth of native tissue, thereby maintaining structural integrity during the critical phases of healing. The degradation of P4HB occurs via hydrolytic and enzymatic pathways. This degradation results in the formation of biocompatible byproducts that are ultimately metabolized and cleared by the body, minimizing the risk of chronic foreign body reactions [16].

A unique feature of PhasixST™ mesh is the incorporation of a non-adhesive barrier on one side, which is designed to minimize tissue attachment and adhesion formation to adjacent viscera. Another key advantage over permanent synthetic meshes, which are more prone to long-term complications, is the mesh’s ability to support tissue regeneration while minimizing chronic inflammation and fibrosis.

## 3. Surgical Technique

Achieving optimal visualization of the hiatus and surrounding structures is essential for precise dissection, safe mesh placement, and effective hernia reduction. The surgical procedure begins with careful patient positioning in Lloyd Davies, typically in a reverse Trendelenburg orientation, which utilizes gravity to facilitate downward displacement of the abdominal viscera, thereby providing better exposure of the esophageal hiatus. Access into the peritoneal cavity is established through Palmer’s point using a 5-mm blunt trocar. The abdomen is insufflated with carbon dioxide gas to a pressure of 15 mmHg. Once the abdomen is insufflated, the following trocars are placed: an 11-mm optical trocar for the 30-degree camera in the midline above the umbilicus, a 5-mm in the right anterior axillary line used for liver retraction, a 5-mm working trocar in the right upper quadrant, and an 11-mm working trocar in the left upper quadrant. The aim of this arrangement is to provide triangulation for effective dissection and suturing. A Diamond flex 80 mm angled liver retractor is commonly used to elevate the left lateral segment of the liver, thereby providing unobstructed access to the diaphragmatic crura.

**Figure 1:**
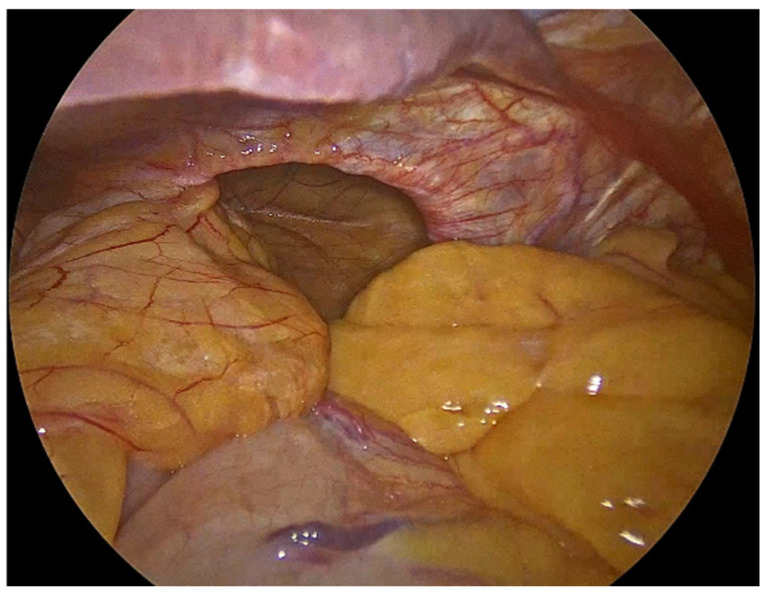
Herniated abdominal viscera and fat through large hiatal defect.

**Figure 2:**
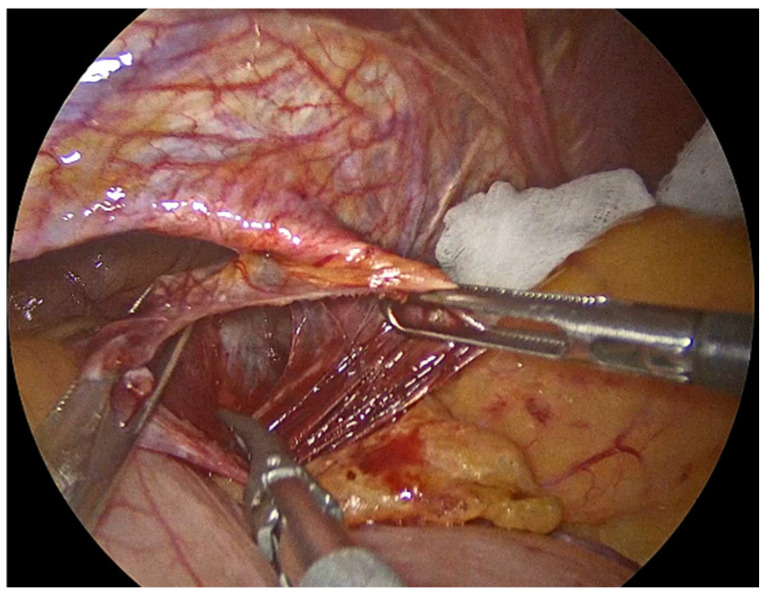
Meticulous separation of the hernia sac from the mediastinum and surrounding tissues.

**Figure 3:**
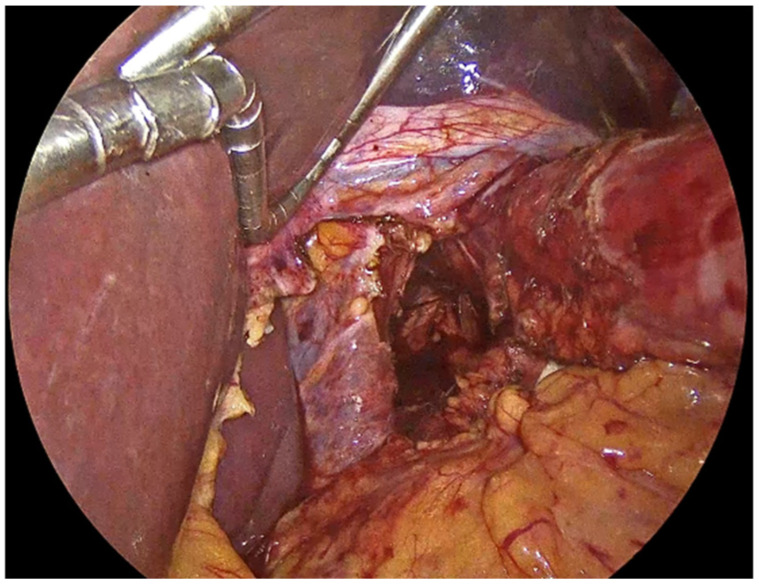
Preservation of the fascial coverings over the hiatal pillars.

The process begins with the complete reduction in herniated abdominal viscera and fat from the mediastinum back into the abdominal cavity (Figure 1). This manoeuvre is essential to restore normal anatomy and to facilitate next steps, including mobilizing the esophagus to achieve adequate intra-abdominal length, mesh placement and crural closure [4,17]. It requires careful separation of the hernia sac from the mediastinum and surrounding tissues using sharp and blunt dissection under direct visualization (Figure 2). The rationale for complete sac dissection is multifaceted: it allows for better visualization of the hiatal anatomy, reduces the risk of residual sac acting as a lead point for recurrence, and facilitates tension-free crural closure [4,18]. Preservation of the fascial coverings over the hiatal pillars during this step is a critical technical detail, as it maintains the integrity of the crura and provides a robust substrate for mesh reinforcement and a durable repair (Figure 3). To reduce the risk of nerve injury and its potential functional consequences, we visualize and preserve both vagal nerves during the procedure. In cases where a retro-cardial lipoma is present, we consider beneficial its mobilization and resection, as it may contribute to the hernia’s bulk and impede complete reduction. The fundus of the stomach is then mobilized upon entry into the abdominal cavity and the short gastric vessels and gastrosplenic ligament are divided along the greater curvature of the stomach until reaching the meeting point of the left and right gastroepiploic arteries.

**Figure 4:**
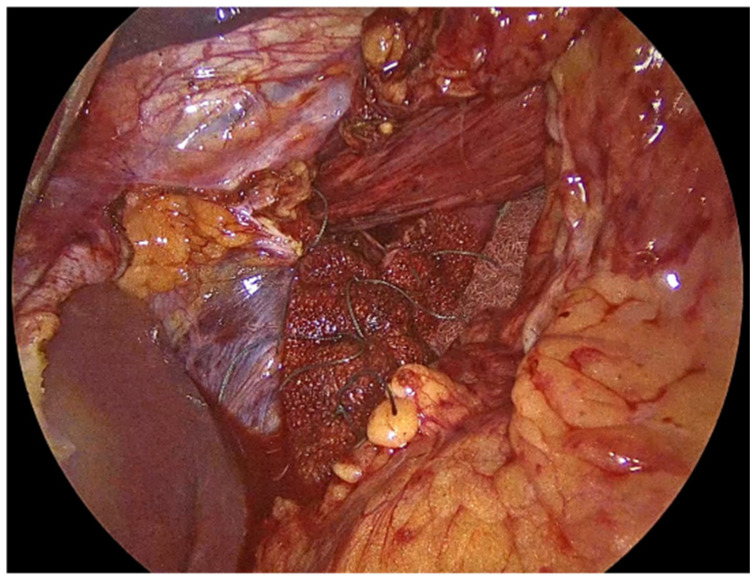
PhasixST™ mesh formed into pledgets which are employed beneath the interrupted sutures to reinforce the suture line during approximation of the crural pillars.

Our primary objective is to achieve a tension-free closure of the diaphragmatic crura, thereby restoring the normal anatomy of the esophageal hiatus and minimizing the risk of hernia recurrence. We emphasize reduction in the hernia sac from the mediastinum and mobilization of at least 2–3 cm of intra-abdominal esophagus as insufficient mobilization may place excessive tension on the repair. The mobilization of the esophagus is critical for creating a retroesophageal window, which facilitates the subsequent steps of mesh placement and crural reinforcement. During this phase, we take care to avoid injury and protect the anterior and posterior vagus nerves, as inadvertent damage can lead to significant morbidity [5,19]. In the following step, we perform meticulous approximation of the crural pillars without undue tension. To do so, we place 3 or 4 single interrupted sutures intracorporeally, depending on the size of the hiatus. We always use No2-0 Ethibond non-absorbable sutures for their long-term durability, as absorbable materials may lose tensile strength before adequate tissue healing has occurred [3]. In some cases, we partially cut the PhasixST™ mesh to form pledgets which are employed beneath the interrupted sutures to reinforce the suture line, particularly in patients with attenuated or fragile crural tissue in order to enhance the strength of the repair (Figure 4) [20]. At this stage of the procedure a 36-French orogastric tube is inserted and advanced to the body of the stomach. It is used to delineate the esophagus from the surrounding tissues and to calibrate the esophageal hiatus and ensure that the closure is neither too loose nor too constrictive.

**Figure 5:**
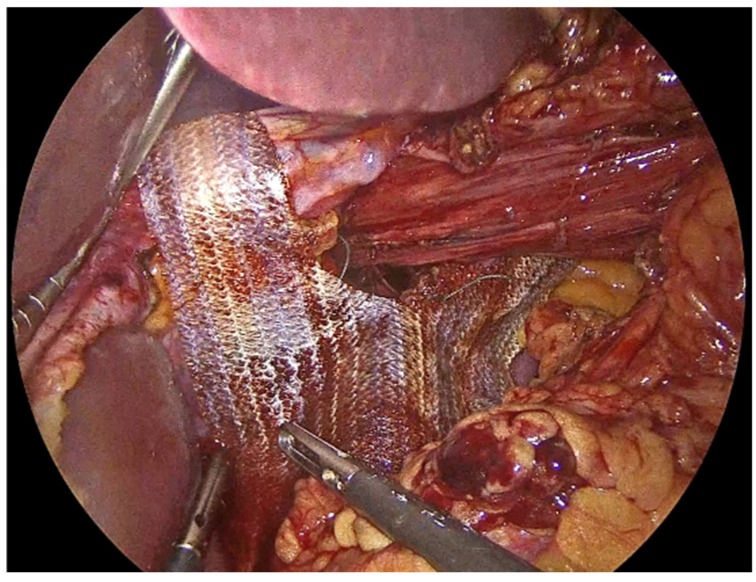
PhasixST™ mesh fashioned into a U-shape configuration to encircle the esophagus without causing constriction.

**Figure 6:**
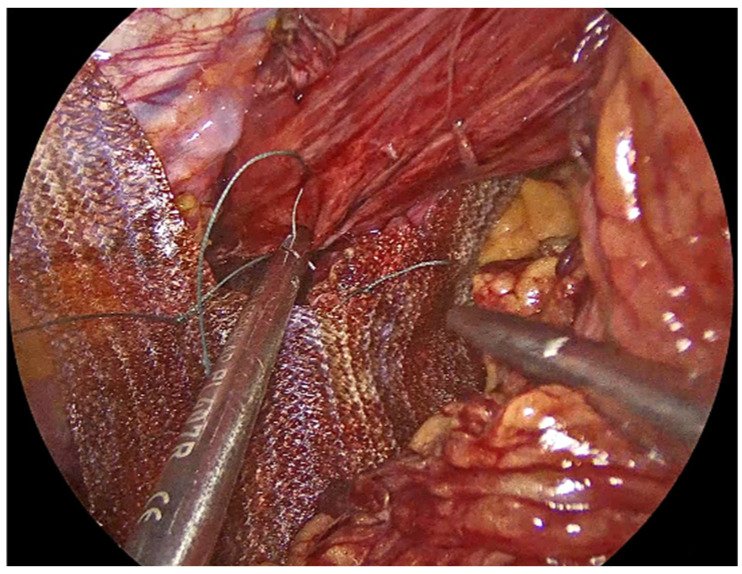
Fixation of the PhasixST™ mesh with interrupted No2-0 Ethibond sutures, anchoring the mesh securely to the diaphragmatic crura.

Once the crura are adequately prepared, the rest of the PhasixST™ mesh is tailored to fit the hiatal defect. The mesh is typically fashioned into a U-shape configuration, allowing it to encircle the esophagus without causing constriction or impeding esophageal motility (Figure 5) [13,21]. The mesh should be positioned posteriorly and laterally to reinforce the crural closure, while care is taken to avoid direct contact with the esophagus to minimize the risk of erosion or stricture formation [19,22]. Ensuring an appropriate overlap of the mesh over the crural closure is essential to provide sufficient reinforcement and to reduce the risk of recurrence [13,21,22]. Fixation of the PhasixST™ mesh is performed with interrupted No2-0 Ethibond non-absorbable sutures, anchoring the mesh securely to the diaphragmatic crura (Figure 6).

**Figure 7:**
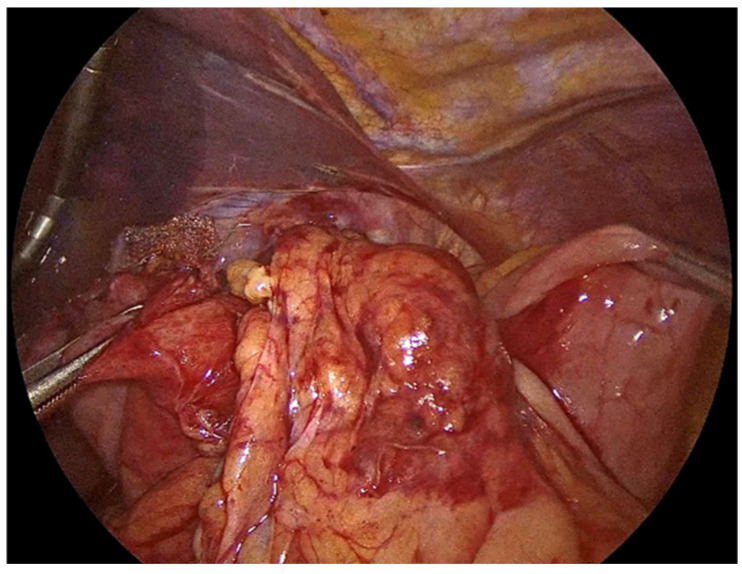
Shoe-shine manoeuvre during Nissen fundoplication.

**Figure 8:**
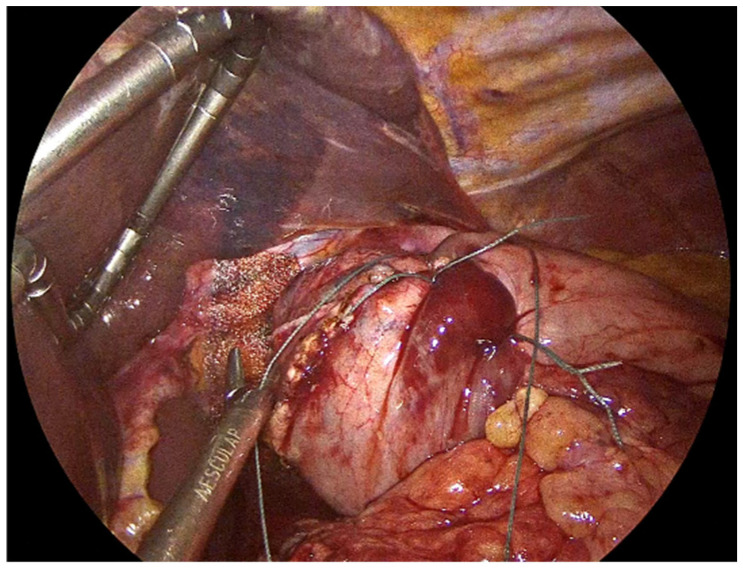
Three interrupted No2-0 Ethibond sutures to complete the Nissen fundoplication and the PhasixST™ covering the diaphragmatic crura.

The hiatal hernia repair is then completed with fundoplication, aiming to restore the antireflux barrier and prevent postoperative gastroesophageal reflux. Several techniques have been developed, each with distinct technical considerations and implications for patient outcomes. The most widely recognized approaches include total (Nissen) fundoplication and partial fundoplication, which can be performed either posteriorly (Toupet, 270°) or anteriorly (Dor, 180°). We always perform Nissen fundoplication which involves a 360° wrap of the gastric fundus around the distal esophagus. The shoe-shine manoeuvre is applied to avoid twisting of the gastric fundus during the fundoplication (Figure 7). Three interrupted No2-0 Ethibond non-absorbable sutures are used to secure the Nissen fundoplication in place (Figure 8). The previously inserted orogastric tube and division of the short gastric vessels provides sufficient laxity for the fundoplication, allowing for a tension-free 360° wrap. Intraoperative testing of the repair using a bougie dilator, allows us to assess the adequacy of the hiatal closure and to detect any undue narrowing that could lead to postoperative stenosis or dysphagia.

**Figure 9:**
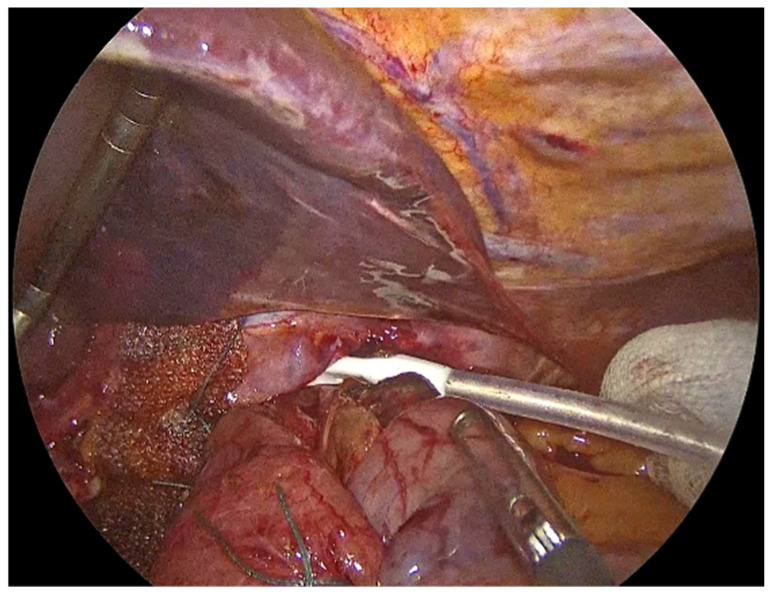
Placement of a drain posteriorly to the fundoplication and through the hiatus.

The surgical procedure is then completed with the placement of a pen-rose drain posteriorly to the fundoplication (Figure 9). Thorough hemostasis is performed, pneumoperitoneum is ceased, and a Valsalva manoeuvre is applied.

Patients are generally started on a liquid diet the first postoperative day, progressing as tolerated to minimize strain on the hiatal repair and reduce the risk of early recurrence or mesh displacement. The transition to a regular diet is guided by the absence of dysphagia, nausea, or vomiting, and is often individualized based on patient recovery and intraoperative findings.

## 4. Discussion

Recent years have witnessed significant progress in mesh technology for hiatal hernia repair, with a particular focus on the development of bioabsorbable and biologic materials designed to address the limitations of traditional permanent synthetic meshes. The evolution of mesh design is driven by the need to reduce mesh-related complications such as erosion, infection, and chronic pain, which have historically been associated with synthetic materials [20].

Gerdes et al. highlight that consensus among experts regarding the optimal approach to hiatal closure is limited, reflecting both the complexity of the condition and the historical reliance on individualized surgical judgment [4]. Complications associated with traditional suture repair are generally related to the technical aspects of the procedure. These may include esophageal or gastric injury, postoperative dysphagia due to overtightening of the hiatus, and, less commonly, suture-related erosion or migration. However, the absence of foreign material in the repair eliminates the risk of mesh-specific complications such as erosion, infection, or chronic inflammation.

The integration of bioabsorbable PhasixST™ mesh into laparoscopic repair of large hiatal hernias has several implications for clinical practice, particularly in the context of balancing recurrence prevention with the minimization of mesh-related complications. Traditional use of permanent synthetic meshes in hiatal hernia repair has been associated with a reduction in recurrence rates, yet this benefit is counterbalanced by the risk of severe complications such as mesh erosion, stricture, and dysphagia, which can significantly impact patient outcomes and quality of life [12,15,23]. The true incidence of these adverse events remains uncertain due to the predominance of short-term follow-up in many studies and inconsistent reporting of complications [12,15].

Studies have demonstrated that even with meticulous suture technique, recurrence rates can reach up to 15% or higher over long-term follow-up, and while many of these recurrences may be radiographic and asymptomatic, there remains concern for progressive symptom development over time [12,20]. Early clinical data suggest that bioabsorbable mesh reinforcement can reduce the incidence of radiographic and symptomatic recurrence compared to suture-only repairs, particularly in patients with large hiatal defects [1,24]. In a comparative study, the recurrence rate in the suture-only group was 18.8% at 12–36 months, whereas the group with mesh reinforcement had no recurrences, indicating a potential benefit of mesh augmentation in high-risk cases [24]. Meta-analyses have shown similar rates of dysphagia between mesh and suture groups, with no statistically significant difference, suggesting that the addition of mesh does not compromise esophageal function in the majority of patients [17]. Studies have reported that both suture-only and mesh-reinforced repairs can lead to significant improvements in GERD-related quality of life scores postoperatively, but the durability of these improvements may be greater in the mesh group due to lower recurrence rates [1,20].

Bioabsorbable meshes like PhasixST™ are designed to provide temporary reinforcement during the critical period of tissue healing, after which the material is gradually resorbed, potentially reducing the long-term risk of mesh-related complications. Deeken et al. demonstrated that the burst strength of PhasixST™ mesh repairs remains stable over time, suggesting that the mesh provides adequate support during the healing phase before absorption [16]. This property is particularly relevant in the context of large hiatal hernias, where the risk of recurrence is elevated due to the inherent weakness of the diaphragmatic crura and the mechanical stress at the gastroesophageal junction [23]. The clinical adoption of PhasixST™ mesh may therefore offer a compromise between the durability of repair and the safety profile of the procedure.

While some studies have shown that mesh reinforcement, in general, can lower recurrence rates compared to suture repair alone, the evidence is not unequivocal, and the optimal technique remains a subject of debate [15,23]. Angeramo and Schlottmann found that mesh reinforcement did not significantly reduce early or late recurrence rates compared to suture repair, and noted an increase in overall morbidity, particularly with nonabsorbable meshes [25]. This highlights the importance of mesh selection, as bioabsorbable options may mitigate some of the risks associated with permanent materials.

While short-term data suggest that mesh reinforcement, especially with biologic or absorbable materials, may lower recurrence rates compared to suture repair alone, the durability of this benefit is uncertain. Westcott et al. highlight that only one study has provided long-term data on absorbable mesh, revealing high and comparable recurrence rates in both mesh and non-mesh groups after five years [20]. This raises questions about whether the initial reduction in recurrence translates into sustained clinical benefit.

In clinical practice, the decision to use mesh, and specifically a bioabsorbable mesh like PhasixST™, should be individualized based on patient factors such as hernia size, tissue quality, comorbidities, and the risk of recurrence. Laxague et al. reported favorable outcomes with mesh reinforcement in redo hiatal hernia repairs, with low rates of re-recurrence and no mesh-related complications in the short- and mid-term [26]. However, the lack of long-term data and the variability in mesh types and surgical techniques underscore the need for further research and standardized protocols [12,13].

The heterogeneity in surgical technique and patient selection further complicates the interpretation of outcomes. Factors such as the method of mesh placement, fixation, and the anatomical characteristics of the hernia can influence both recurrence and complication rates [27,28]. Surgeons must also consider the technical aspects of mesh placement, as improper positioning or fixation can contribute to complications or recurrence. The literature suggests that careful intraoperative assessment of the crural defect and tailored mesh application are critical for optimizing outcomes [26]. Additionally, the use of bioabsorbable mesh may be particularly advantageous in high-risk or elderly patients, where the avoidance of permanent foreign material is desirable due to the increased susceptibility to complications [15,26].

A critical analysis of the current literature on bioabsorbable PhasixST™ mesh for laparoscopic repair of large hiatal hernias reveals several limitations that must be addressed to accurately interpret the available evidence. One of the most significant constraints is the heterogeneity in study design and methodology. Many investigations rely on retrospective analyses or non-randomized cohorts, which inherently introduce selection bias and limit the strength of causal inferences that can be drawn regarding mesh efficacy and safety. Antoniou et al. state that even when randomized controlled trials are included, sensitivity analyses often reveal that the short-term benefits observed may not persist, and the risk of bias remains a concern due to the variability in study protocols and patient populations [29].

Another limitation is the relatively short duration of follow-up in most studies. The mean objective follow-up periods often range from only several months to a few years, which is insufficient to fully capture the long-term recurrence rates and potential late complications associated with bioabsorbable mesh use. Latorre-Rodríguez et al. highlight that while some mesh brands demonstrate low recurrence rates at intermediate follow-up, the absence of extended longitudinal data precludes definitive conclusions about durability and late adverse events [28].

The lack of standardized outcome measures further complicates the interpretation of results. Studies frequently employ different definitions for hernia recurrence, mesh-related complications, and clinical success, making direct comparisons challenging [28,30]. For instance, some studies define recurrence radiologically as any portion of the stomach above the diaphragm, while others require a minimum hernia size, such as greater than 2 cm, to classify as recurrence [31]. This heterogeneity complicates direct comparison across studies and underscores the need for standardized definitions and imaging protocols. Manara et al. emphasize that the diversity in search strategies and inclusion criteria across systematic reviews and meta-analyses can lead to inconsistent quality assessments and variable reporting of outcomes [30].

The degree of mesh contact with the esophagus, the technique of fixation, and whether the mesh completely encircles the esophagus are all factors that influence the likelihood of complications. Inadequate fixation can lead to mesh migration, while excessive contact or circumferential placement increases the risk of erosion and stricture formation. The literature indicates that while the use of bioabsorbable mesh is intended to mitigate the long-term risks associated with permanent prosthetics, such as erosion and chronic infection, perioperative complications are not entirely eliminated [7,14]. The most feared adverse event associated with mesh reinforcement is erosion into the esophagus or stomach, which can result in fistula formation, stenosis, and even cardiac tamponade in rare cases. The literature indicates that mesh-related complications, while relatively infrequent, are not negligible. A meta-analysis encompassing 5499 laparoscopic paraesophageal hernia repairs with mesh reinforcement reported a 1.9% incidence of mesh-associated complications, with esophageal and gastric erosions being the most prevalent, followed by stenosis and other serious outcomes. Notably, both synthetic materials such as polypropylene and PTFE, as well as biologic meshes, have been implicated in these adverse events, though the rates may vary depending on the material properties and patient selection [7]. Dysphagia remains a notable concern in the immediate postoperative period. Even with biological mesh, postoperative dysphagia has been reported in 12–17.5% of patients, suggesting that the introduction of mesh, regardless of its absorbability, can contribute to transient or persistent swallowing difficulties [14].

Another important consideration is the underreporting of technical variables, such as mesh sizing, fixation methods, and surgeon experience, all of which can influence outcomes but are inconsistently documented in the literature [32]. The variability in surgical technique, including the approach to crural reinforcement and the management of adhesions, introduces additional confounding factors that are rarely controlled for in existing studies [27].

The interpretation of the current literature on mesh reinforcement in large hiatal hernia repair reveals a nuanced and evolving landscape. However, robust comparative data specifically evaluating PhasixST™ or similar bioabsorbable meshes in large hiatal hernia repair are still lacking. Whitehead-Clarke et al. underscore the importance of ongoing post-market surveillance and the development of hernia registries to capture real-world outcomes, particularly as new mesh materials are introduced [33]. The lack of standardized, long-term data collection for hiatal hernia devices limits the ability to assess the true impact of innovations such as PhasixST™ on patient outcomes. Roth et al. and Campos et al. both emphasize the need for standardized operative techniques and consistent postoperative follow-up to enable meaningful comparisons across studies [34,35].

In summary, the literature suggests that while mesh reinforcement, whether permanent, biologic, or bioabsorbable, may offer some short-term benefits in reducing recurrence after large hiatal hernia repair, the evidence for long-term superiority, particularly for bioabsorbable meshes like PhasixST™, remains inconclusive. The potential for reduced mesh-related complications with bioabsorbable materials is an attractive hypothesis, but definitive data are lacking. Ongoing surveillance, high-quality RCTs with extended follow-up, and standardized outcome reporting are essential to clarify the role of PhasixST™ and similar meshes in this context [15,36,37].

## 5. Conclusions

The current literature underscores the evolving landscape of large hiatal hernia repair, particularly with the integration of bioabsorbable meshes such as PhasixST™. These meshes, composed of poly-4-hydroxybutyrate, offer a promising alternative to permanent synthetic materials by providing prolonged mechanical support during the critical healing phase while gradually resorbing to minimize long-term foreign body complications. Clinical data suggest that PhasixST™ mesh may reduce short- to mid-term recurrence rates compared to suture-only repairs and biological meshes, with a favourable safety profile characterized by a low incidence of mesh-related complications such as erosion, infection, and chronic pain. The biochemical and biomechanical properties of P4HB contribute to its suitability for crural reinforcement, balancing tensile strength, flexibility, and biocompatibility to support tissue integration and remodelling.

From a clinical perspective, the adoption of bioabsorbable mesh reinforcement should be guided by a careful assessment of individual patient risk factors and anatomical considerations. The potential to reduce recurrence without increasing mesh-related morbidity is particularly relevant in high-risk populations, including those with large or recurrent hernias and compromised tissue quality. Meticulous surgical technique, including complete hernia sac dissection, adequate esophageal mobilization, tension-free crural closure or bioabsorbable mesh reinforced cruroplasty and precise mesh placement, remains paramount to achieving optimal outcomes. Postoperative management protocols emphasizing early detection of complications, structured follow-up with symptom assessment and imaging, and patient education are essential components of comprehensive care.

In summary, PhasixST™ mesh represents a promising advancement in the pursuit of more durable and safer hiatal hernia repairs. By providing temporary reinforcement during the period of greatest biomechanical demand while reducing the long-term risks associated with permanent implants, PhasixST™ may help achieve an optimal balance between repair durability and patient safety. Continued research, particularly through well-designed, multicenter randomized controlled trials with long-term follow-up, is essential to validate these early findings and further refine surgical practice to improve patient outcomes.

## 6. Future Directions

Despite the growing interest in bioabsorbable meshes such as PhasixST™ for laparoscopic repair of large hiatal hernias, several significant gaps in the current evidence base remain. One of the most serious limitations is the lack of high-quality, long-term comparative data evaluating bioabsorbable meshes against both permanent synthetic and biologic alternatives. The majority of available studies focus on short- to mid-term outcomes, with limited follow-up durations that do not adequately capture late recurrences or mesh-related complications [15,24,38]. This shortcoming is particularly relevant given that some mesh materials, including P4HB, are designed to resorb over 12–18 months, and thus, their true impact on tissue remodeling and hernia durability may only become apparent after several years [32].

Future research should prioritize large, multicenter long-term randomized controlled trials with standardized outcome measures, extended follow-up, and robust reporting of both efficacy and safety endpoints [15,24,38,39].

## Abbreviations

The following abbreviations are used in this manuscript:

P4HB	Poly-4-hydroxybutyrate
GERD	Gastroesophageal reflux disease
GEJ	Gastroesophageal junction
PTFE	Polytetrafluoroethylene
RCTs	Randomized control trials
